# mHealth Apps Assessment among Postpartum Women with Obesity and Depression

**DOI:** 10.3390/healthcare8020072

**Published:** 2020-03-26

**Authors:** Che Wan Jasimah Bt Wan Mohamed Radzi, Hashem Salarzadeh Jenatabadi, Nadia Samsudin

**Affiliations:** Department of Science and Technology Studies, Faculty of Science, University of Malaya, Kuala Lumpur 50603, Malaysia; jasimah@um.edu.my (C.W.J.B.W.M.R.); nadiasamz91@gmail.com (N.S.)

**Keywords:** mHealth apps, postpartum obesity, postpartum depression, health assessment

## Abstract

Background: Pregnancy has become the main constituent for women to become overweight or obese during the postpartum phase. This could lead women to suffer from postpartum depression as well. Information technology (IT) has become more prevalent in the healthcare industry. It offers patients the opportunity to manage their health conditions via the use of several applications, one being the mHealth applications. Objective: The main purpose of this study is to experiment and understand the effects the mHealth applications (i.e., fitness and nutrition applications) have on the body mass index (BMI) and depression levels amongst postpartum women. Methods: Online questionnaires were sent to postpartum women within one year after their pregnancy, of which 819 completed questionnaires were returned. The frequency of the mHealth applications usage was categorized into daily, weekly, rarely and never streams. Therefore, the frequency of use of the mHealth applications for BMI and depression levels was analyzed based on the available statistical data. Descriptive statistics, ANOVA, and Dunnet tests were applied to analyze the experimental data. Results: Out of 819 respondents, 37.9% and 42.1% of them were overweight and obese, respectively. Almost 32.9% of the respondents were likely depressed, and 45.6% were at an increased risk. This study reports that only 23.4% and 28.6% of respondents never used the fitness and nutrition applications. The impact of the frequency of using the fitness applications on BMI and depression levels was obvious. This means that with the increased use of the fitness applications, there was also a significant effect in maintaining and decreasing the BMI and depression levels amongst Malaysians postpartum women. However, from the data of weekly and daily use of fitness applications, we found that the contribution toward the BMI and depression levels was high (*p* = 0.000). However, nutrition applications amongst the users were not significant within the main variables (*p* > 0.05). From the Dunnet test, the significance of using the fitness applications within the depression levels started from daily usage, whereas for BMI, it started from weekly usage. Conclusion: The efficiency of the fitness applications toward the BMI and depression levels has been proven in this research work. While nutrition applications did not affect the BMI and depression levels, some of the respondents were still categorized as weekly and daily users. Thus, the improvements in BMI and depression levels are associated with the types of mHealth app that had been used.

## 1. Introduction

mHealth has been identified as a public medical and health application which is to be paired with mobile devices [1]. The development of mobile communication devices (e.g., smartphones, tablets, etc.) has resulted in many creative innovations with regard to mobile applications (apps), especially in the healthcare industry, which is also known as the mHealth app [2]. The increasing number of apps in the App Store shows that mHealth apps are available to be downloaded by anyone at any time [3]. Prior studies have focused on mHealth apps for health purposes and the potential benefits in precautionary health, including healthcare and the challenges of using the apps [4,5]. Cho, Lee, Kim and Park [6] reported that the main reasons for downloading mHealth apps were for diet/calorie counting and exercise/fitness. Studies have shown that the fitness, nutrition and behavior apps were the most commonly downloaded mHealth apps [7,8]. Basically, fitness and nutrition apps may help in promoting a healthy lifestyle [9,10]. The fitness apps promoted weight loss, healthier eating, higher exercise levels, and lower BMI levels, whereas the nutrition apps were correlated with better food intake control and weight loss [9].

Despite the rapid download rate, Peng, Kanthawala, Yuan, and Hussain [11] mentioned that many mHealth apps did not get sufficient feedback from the users. Therefore, if the apps did not reach their expectations, they tended to uninstall it. These days, women spend more time using smartphones compared to men [12]. Goh et al. [13] demonstrated that women happened to be more likely to download health and fitness apps on their smartphone. Nevertheless, the study on postpartum women associated with mHealth apps seems insufficient. Postpartum-related mHealth intervention has been investigated and was related to postpartum family planning amongst couples [14,15,16] and HIV prevention [17]. Moreover, globally, one-third of adult women suffer from obesity [18,19]. As the years pass, the risk of obesity amongst women has aggressively increased [20]. In addition, Keshavarz et al. [21] and Rogan, Payne, and Meltzer-Brody [22] have proven the connection between postpartum depression and obesity. This claim has also been supported by Sumithran et al. [23], whereby the connection was quite prevalent amongst postpartum women. During the postpartum period, one in seven women faces depression due to complications that plague them after childbirth [24]. Apparently, more than 20% of women have been diagnosed with having postpartum depression [25].

mHealth apps related to weight management [26] and mental health or depression treatment [27] have already been used widely in recent years. However, in this study, we focus on only two mHealth apps related to fitness and nutrition to see if they can affect postpartum obesity and depression. To the best of our knowledge, the number of studies on the use of fitness and nutrition apps and their relationship with BMI and depression levels amongst postpartum women is scarce. Therefore, we conducted this study to evaluate the impact of fitness and nutrition apps on postpartum women with obesity and depression. This would offer a much better understanding on which factors are associated with the use of these apps.

## 2. Materials and Methods 

### 2.1. Participants 

In determining the sample size for this research, we used Krejcie and Morgan’s method to calculate the required sample size [28]. The given formula has commonly been referred in estimating the sample size [29]:

s = X^2^NP (1 − P) / d^2^(N − 1) + X^2^P(1 − P) (1)

where

s = the required sample size
X^2^ = the table value of chi-square for one degree of freedom at the desired confidence level
N = the population size
P = the population proportion
d = the degree of accuracy expressed as a proportion (0.05)

This research focused on Malaysian postpartum women who live in urbanized and populated cities due to the easy access to the internet. The subjects were chosen from around Kuala Lumpur, Johor, Penang and Selangor. The data were distributed via online questionnaires, by sending the link of the questionnaires to the participants. Therefore, our sample structure was concerned with participants who had access to the internet. Moreover, this research was about the impact of mHealth application on women with depression and obesity. Subsequently, postpartum women who did not have access to the internet were automatically eliminated from our research sampling.

The target group was in the one-year postpartum period after their pregnancy. We chose this period of time because it is a suitable period, as previous studies [30,31] claimed mothers usually recover from childbirth in terms of physical and mental factors. We received 819 completed questionnaires from the respondents. 

### 2.2. Ethics Statement

The survey was conducted with the approval from the University of Malaya’s Research Ethics Committee (UM.TNC2/RC/H&E/UMREC 127). The research methods were performed in accordance with the relevant guidelines and regulations. Respondents were provided with an explanation of the research purpose and informed consent was obtained from all respondents along with the online questionnaire. 

### 2.3. Measurement 

The research variables found were classified into four sections: demographics, BMI, depression level, and frequency of use of mHealth apps. 

Demographics. Four indicators, i.e., age group, education background, work experience and income household per month, were included in the demographics variables. The age range was divided into four groups: 21 to 25 years old, 26 to 30 years old, 31 to 35 years old and over 35 years old. The education background was denoted as “Less than high school”, “High school”, “Diploma”, “Bachelor” and “Master or Ph.D.”. The work experience of the respondents was categorized as “No job experience”, “less than 3 years”, “3 to 6 years”, “6 to 10 years”, and “More than 10 years”. The household income per month in Ringgit Malaysia (RM) was divided into “Less than RM 2000”, “RM 2000–RM 3000”, “RM 3000-RM 4000”, “RM 4000–RM 5000”, and “Over RM 5000”.

BMI. To measure the BMI range of the individuals, height and weight needed to be calculated using the standardized formula: [Weight in kilograms] / [Height in meters]^2^ [32,33]. The BMI categories were underweight (<18.5 kg/m^2^), normal (18.5 to 23.9 kg/m^2^), overweight (24.0 to 27.9 kg/m^2^), and obese (≥28.0 kg/m^2^) [34].

Depression. Depressive symptoms were measured using the Edinburgh Postnatal Depression Scale (EPDS) questionnaire, as it was validated during the prenatal and postpartum periods. It has also been used in previous studies [35,36,37,38,39]. The EPDS was calculated using a four-point scale for ten items in the questionnaire to measure the frequency of the depressive symptoms. A total score of 0–9 was categorized as “normal”, scores of 10–11 were categorized as “slightly increased risk”, scores of 12 to 15 as “increased risk” and those more than 15 were listed as “likely depression” [40].

mHealth apps frequency use. Respondents were asked about their status of using the apps, either “Yes” or “No” for using the apps. Regarding how long they had been using the apps, the participants were categorized into four groups denoted as “less than 1 month”, “1–2 months”, “3–5 months”, and “more than 5 months”. Meanwhile, the frequency of the respondents using the fitness and nutrition apps was categorized into four groups, indicated as “never used”, “rarely”, “weekly” and “daily”, which has also been used in previous studies [41]. Those respondents who used the apps less than 1 month were considered as having never used the apps. We clarified the types of app that had commonly been installed, including those for finding information (e.g., exercise guidelines), tracking diet/fitness activities (e.g., daily history), and interacting with users (e.g., feedback) [42].

### 2.4. Data Analysis

Descriptive statistics of the research variables were tabulated according to the distribution of the research participants. The main variables (i.e., BMI, depression level, frequency of fitness and nutrition apps use) were compared for the distribution of the participants, as it can be analyzed based on the purpose of the research work. Thus, a one-way analysis of variance (ANOVA) to analyze the impact of the mHealth apps frequency use on postpartum BMI and depression was obtained. The analysis of the data continued with the Dunnett t-tests. The test has the ability to treat one group as a control and compare all other groups against it. All P-values that obtained less than 0.05 were considered to be statistically significant. Analyses were performed using SPSS (version 25, SPSS Inc., Chicago, IL, USA).

## 3. Results

Table 1 presents the descriptive statistics on the research variables. Among 819 postpartum women (majority aged 31 years and older), 340 of them (41.5%) were Malay, 295 (36.0%) were Chinese, and 184 (22.5%) were Indian. Most of participants possessed a Bachelor’s degree (34.2%) and 28.9% were Diploma holders. Only 133 (16.2%) of the respondents did not have any job experience and most of them had 7 to 10 years of work experience (28.1%). In terms of the highest monthly income, 365 (44.6%) of respondents received around RM 4000 to 5000 per month, and only 36 (4.4%) received over RM 5000 per month. With regard to the BMI variable, the fewest number of respondents were recorded as underweight (12.0%) and most were obese (32.0%). A total of 223 respondents (27.2%) were of normal weight, and 236 (28.8%) were overweight out of the 819 postpartum women studied. From the calculated EPDS score, only 18.9% of the respondents were normal. Depression levels for the rest of the respondents were 21.4% (slightly increased risk), 34.7% (increased risk), and 25% (likely depression). Meanwhile, the average frequency of the fitness apps’ use was 2.53 (towards weekly usage). The frequency of weekly use of the fitness apps was the highest at 28.0% (229/819) respondents; 24.4% (200/819) and 24.2% (198/819) of respondents used the fitness apps daily and rarely, respectively, while 23.4% of them (192/819) never used the fitness apps. On the other hand, for nutrition apps, 29.2% (239/819) of respondents rarely used the apps, followed by the “never” category at 28.6% (234/819). The rest of the respondents used nutrition apps almost weekly and daily by 23.2% (190/819) and 19.0% (156/819), respectively. The average frequency of the use of nutrition apps was at 2.33 (towards rare usage).

The distribution of the BMI and depression levels across different frequencies of using fitness and nutrition apps is illustrated in Table 2 and Table 3, respectively. Based on Table 2, “never use” and “rarely use’ regarding the fitness apps formed the majority of the respondents who were overweight and obese. Most of the weekly and daily users of the fitness apps were in the normal BMI category. For respondents who never used the fitness apps, only 16.1% of them were of normal BMI and devoid of depression symptoms. The “rarely” fitness apps users only constituted 15.2% of the respondents and 16.2% of weekly users who did not show depression symptoms. Nevertheless, 19.5% of the daily users of the fitness apps were likely depressed.

From Table 3, “never use” and “rarely use” regarding the nutrition apps formed the majority of the respondents who were overweight and obese, respectively. However, most of the weekly and daily users of the nutrition apps were in the normal and obese BMI categories, respectively. Only 17.1% of the respondents who never used the nutrition apps, 15.9% of the rare users, 21.6% of the weekly users, and 23.1% of the daily users were reported to have shown no depression symptoms.

We present the ANOVA output in Table 4. From the output, the differences between the significant results of the frequency of using the fitness and nutrition apps were observable. The BMI and depression levels for the fitness apps achieved a p-value of less than 0.05. Moreover, the p-value obtained for the nutrition apps for BMI and depression levels were higher than 0.05 (0.204 and 0.094, respectively). Therefore, we performed the post hoc tests using the Dunnet t-test to compare the frequency of one group with that of another group. Based on Table 5, the “never use” group was kept as the control group and all other groups were compared against it. As for the BMI variable, comparisons between the frequency of the use of the fitness apps for the weekly (*p*-value < 0.01) and daily (*p*-value < 0.01) groups were significant, but not significant with the rarely group (*p*-value = 0.197). Nevertheless, for the depression level variable, the only significant comparison was between the “daily” and “never use” fitness apps users (*p*-value < 0.01). With regard to the Dunnet t-test on the nutrition apps frequency, all comparisons between the groups were less significant, regardless of whether it was related to the BMI or depression level variables (*p*-value > 0.05).

## 4. Discussion

This study investigated how frequently fitness and nutrition apps were used, which can affect BMI and depression levels. The results illustrated that the prevalence of overweight and obesity factors amongst postpartum women in the research sample was quite high. The main purpose of this research was achieved across three phases. In the first phase, descriptive statistics of the postpartum distribution were constructed. The second phase was the analysis of the effects of the frequency of use of fitness and nutrition apps on the BMI and depression levels. The last phase was performed using ANOVA and Dunnet t-tests for both the mHealth apps’ frequency for the comparison analysis within the BMI and depression levels. 

The data were collected from Kuala Lumpur, Johor, Penang and Selangor. The respondents were in the first year of the postpartum period, after their pregnancy, which resembled the research conducted by Kubota et al. [38]. Nevertheless, weight and depression levels are likely to be higher in the first week postpartum compared to one year postpartum [43,44]. However, we recognized the one year postpartum period would be an appropriate time, as mothers usually have recovered both mentally and physically from childbirth by then which has been retained in previous studies [30,31]. During the postpartum period, a factor that contributes to women becoming overweight or obese is an unhealthy lifestyle [45]. It has been reported that many women did not lose the gestation weight gained, which makes it likely for them to be overweight and obese in the postpartum period [46]. Consequently, women are more likely to become obese in the five-year period that follows giving birth once or twice [47]. 

As this study is about postpartum women, it involves a majority group of women who had higher BMI levels due to pregnancy. Nowadays, depression has become a common health problem correlated with obesity [48,49]. It is proven that women who experience excessive weight gain after pregnancy might be at increased risk for postpartum depressive symptoms [50].

The women who participated in this research were measured by EPDS to see their level of depression symptoms. The EPDS scores obtained from the respondents show that many postpartum women need medical check-ups, as most of the respondents were at increased risk and likely depression levels, respectively. Fitness and nutrition apps were familiar to the respondents. From the results in Table 1, the minority of respondents chose “never use” for both apps. In the circle of “never use”, more than half of the respondents were at risk of having depression, and many of them were overweight and obese. For respondents who never used the nutrition apps, they were at risk of having depression and had a higher BMI level. The distribution of the fitness and nutrition apps “rarely” users was slightly higher, which was almost the same as the “never use” respondents. It was also found that the “rarely” users had a higher level of depression, respectively. Moreover, they also had a higher level of BMI. This corresponds to the research of Chan and Chen [51] which proved that the poor use of mHealth apps could lead to low effectiveness regarding weight management and mental health (e.g. depression). 

Apart from that, most of the weekly users of the fitness and nutrition apps were at an increased risk level of depression. Nevertheless, based on the BMI variable, the fitness app users were at the normal level, and fewer respondents were overweight and obese. Meanwhile, for the weekly users of the nutrition apps, the respondents were at the normal BMI levels. In spite of that, the total overweight and obese weekly nutrition app users constituted more than half of the cohort, which was more than the respondents with a normal BMI level. Meanwhile, very few daily users of the fitness apps displayed “likely depression” yet had a higher BMI level. For the daily users of the fitness apps who were overweight and obese, it can be assumed that they had just started to use the apps. The nutrition apps’ daily user distribution did not affect the BMI and depression levels. Even though respondents used the nutrition apps daily, the distribution of the BMI and depression levels were high. This claim was proven based on the ANOVA test obtained from the nutrition apps for the depression levels and BMI p-value which was more than 0.05. Dodd et al. [52] and Olson et al. [53] claimed that low use of mHealth apps affected the effectiveness of the output. However, in this study, we found that other than the frequency of mHealth apps use, the types of app also affected the effectiveness of the app’s intervention in person’s life. 

Therefore, the Dunnet test in Table 5 explains that the significance of using the fitness apps within the depression levels started from the fourth level (i.e., daily use) and BMI starts from the third level (i.e., weekly). The efficiency of the fitness apps for the depression level required more frequent usage of the apps compared to the BMI levels. A more obvious change to the depression levels can be observed if the respondents used the fitness apps daily, while for BMI levels, it would have been more effective if the respondents used the fitness apps weekly. The Dunnet t-test for nutrition apps did not obtain any significant value for every level of the group comparison. In this research, the fitness apps were far more effective than the nutrition apps. Nutrition apps seemed to provide no prominent effects on the depression and BMI levels of the respondents. However, fitness apps do contribute to the depression and BMI levels. Fitness apps can be useful in changing people’s lifestyles by encouraging them to be physically active [54], thus preventing them from having a high BMI level [55]. Thus, physical activity helps people to improve their health, especially in reducing weight or achieving a normal BMI level [56]. As for postpartum depression levels, physical activity has been claimed to be one of the prevention factors [57]. 

However, this research also has a number of limitations. The respondents’ weight and height (to calculate the BMI) were self-reported in this study. Despite previous research works which also used the same method, we considered the data as valid [58,59,60]. Meanwhile, the frequency of using the mHealth apps was also self-reported by the respondents, following the previous research as well [9]. In addition, the validity of the self-reported method on the usage of mHealth apps has already been covered by Robbins et al. [61] and Ruiz et al. [62]. 

On top of that, questionnaires were sent to women who can easily access the internet, and this implies the loss of a significant proportion of women who cannot. As this research is about mHealth applications, therefore postpartum women who lack access to the internet were automatically eliminated from the research sampling. We chose the subjects based on this criterion due to the fact that our survey could only be answered online. In sum, this study is an ICT-based structure, similar to a few other studies [31,63].

The measurement of depression levels using EPDS was also not a substitute for clinical diagnosis. EPDS was used in this research to determine the depression symptoms, if the participants were at risk or normal. Furthermore, it was reported from previous research that many people, especially women, who suffer from depression do not seek medical help [64]. Other than that, there are some other additional treatments may have been received by respondents such as psychiatrists, psychologists, personal trainers, and medicines that may have contributed to the results achieved. Those treatments history might be affecting the BMI and depression levels among respondents. For future study, we suggest the combination of these factors with the intervention of mHealth apps use. 

## 5. Conclusions

With the availability of advanced technology today, mHealth apps help people to manage their lifestyle. People chose to use the mHealth apps for daily routine self-management activities [65], record individual health behavior, track health data, and improve the quality of care [66]. During the postpartum period, mothers are vulnerable to weight gain and are prone to suffer from depression after giving birth. Hence, our research is the first study on postpartum women associated with the frequency of using the mHealth apps by focusing on BMI and depression levels as the main variables. The obesity rate has increased aggressively globally, which has led to fitness apps becoming “self-helps” for users to lose weight [67]. Fitness apps help to encourage people to carry out the right exercise, diet, weight management, stress relief, and sleep monitoring [68]. Our research has proven that the intervention by fitness apps contributes more toward having a better quality of life (in terms of BMI and depression levels) than that by nutrition apps. 

## Figures and Tables

**Table 1:** Descriptive statistics of research variables.

	Number, Percentage
**Age** (*n*, %)	
21 to 25 years old	54 (6.6%)
26 to 30 years old	177 (21.6%)
31 to 35 years old	319 (38.9%)
Over 35 years old	269 (32.8%)
**Education** (*n*, %)	
Less than high school	141 (17.2%)
High school	95 (11.6%)
Diploma	237 (28.9%)
Bachelor	280 (34.2%)
Master or PhD	66 (8.1%)
**Income** (*n*, %)	
Less than RM 2000	144 (17.6%)
RM 2000–RM 3000	124 (15.1%)
RM 3000–RM 4000	150 (18.3%)
RM 4000–RM 5000	365 (44.6%)
Over RM 5000	36 (4.4%)
**Job Experience** (*n*, %)	
No job experience	133 (16.2%)
Less than 3 years	147 (18.0%)
3–6 years	183 (22.3%)
6–10 years	230 (28.1%)
More than 10 years	126 (15.4%)
**BMI** (*n*, %)	
Underweight	98 (12.0%)
Normal	223 (27.2%)
Overweight	236 (28.8%)
Obese	262 (32.0%)
**Depression Level** (*n*, %)	
Normal	155 (18.9%)
Slightly increased risk	175 (21.4%)
Increased risk	284 (34.7%)
Likely depression	205 (25.0%)
**Fitness apps frequency of use** (*n*, %)	
Never used	192 (23.4%)
Rarely	198 (24.2%)
Weekly	229 (28.0%)
Daily	200 (24.4%)
**Nutrition apps frequency of use** (*n*, %)	
Never used	234 (28.6%)
Rarely	239 (29.2%)
Weekly	190 (23.2%)
Daily	156 (19.0%)

**Table 2:** Distribution of BMI and depression in different frequency of using fitness apps.

	BMI (*n*, %)		Depression (*n*, %)	
Never use	192	23.4%	192	23.4%
1	8	4.2%	31	16.1%
2	13	6.8%	35	18.2%
3	86	44.8%	69	35.9%
4	85	44.3%	57	29.7%
Rarely	198	24.2%	198	24.2%
1	15	7.6%	30	15.2%
2	23	11.6%	37	18.7%
3	82	41.4%	72	36.4%
4	78	39.4%	59	29.8%
Weekly	229	28.0%	229	28.0%
1	37	16.2%	37	16.2%
2	92	40.2%	56	24.5%
3	38	16.6%	86	37.6%
4	62	27.1%	50	21.8%
Daily	200	24.4%	200	24.4%
1	38	19.0%	57	28.5%
2	95	47.5%	47	23.5%
3	30	15.0%	57	28.5%
4	37	18.5%	39	19.5%
Grand Total	819	100.0%	819	100.0%

BMI category: 1 = underweight, 2 = normal, 3 = overweight, 4 = obese; Level of depression: 1 = normal, 2 = slightly increased risk, 3 = increased risk, 4 = likely depression.

**Table 3:** Distribution of BMI and depression in different frequency of using nutrition apps.

	BMI (n, %)		Depression (n,%)	
Never use	234	28.6%	234	28.6%
1	33	14.1%	40	17.1%
2	62	26.5%	49	20.9%
3	71	30.3%	82	35.0%
4	68	29.1%	63	26.9%
Rarely	239	29.2%	239	29.2%
1	31	13.0%	38	15.9%
2	50	20.9%	48	20.1%
3	70	29.3%	88	36.8%
4	88	36.8%	65	27.2%
Weekly	190	23.2%	190	23.2%
1	19	10.0%	41	21.6%
2	68	35.8%	44	23.2%
3	49	25.8%	68	35.8%
4	54	28.4%	37	19.5%
Daily	156	19.0%	156	19.0%
1	15	9.6%	36	23.1%
2	43	27.6%	34	21.8%
3	46	29.5%	46	29.5%
4	52	33.3%	40	25.6%
Grand Total	819	100.0%	819	100.0%

BMI category: 1 = underweight, 2 = normal, 3 = overweight, 4 = obese; Level of depression: 1 = normal, 2 = slightly increased risk, 3 = increased risk, 4 = likely depression.

**Table 4:** ANOVA output.

**Frequency of using fitness apps**	**BMI**		**Sum of squares**	**df**	**Mean square**	**F**	**Significant**
		**Between groups**	126.405	3	42.135	47.661	0.000
		**Within groups**	720.499	815	0.884		
		**Total**	846.904	818			
	**Depression level**	**Between groups**	22.267	3	7.422	6.859	0.000
		**Within groups**	882.006	815	1.082		
		**Total**	904.274	818			
**Frequency of using nutrition apps**	**BMI**		**Sum of squares**	**df**	**Mean square**	**F**	**Significant**
		**Between groups**	4.757	3	1.586	1.534	0.204
		**Within groups**	842.147	815	1.033		
		**Total**	846.904	818			
	**Depression level**	**Between groups**	7.066	3	2.355	2.140	0.094
		**Within groups**	897.207	815	1.101		
		**Total**	904.274	818			

**Table 5:** Post Hoc Tests (Dunnet Tests).

**Dependent variable**	**Fitness apps frequency (I)**	**Fitness apps frequency (J)**	**Mean difference (I–J)**	**Std. Error**	**Significant**	**95% Confidence interval**	
						**Lower bound**	**Upper bound**
BMI	Rarely	Never use	−0.165	0.095	0.197	−0.39	0.06
	Weekly	Never use	−0.746 *	0.092	0.000	−0.96	−0.53
	Daily	Never use	−0.962 *	0.095	0.000	−1.18	−0.74
Depression level	Rarely	Never use	0.016	0.105	0.997	−0.23	0.26
	Weekly	Never use	−0.141	0.102	0.366	−0.38	0.10
	Daily	Never use	−0.402 *	0.105	0.000	−0.65	−0.15
**Dependent variable**	**Nutrition apps frequency (M)**	**Nutrition apps frequency (N)**	**Mean difference (M-N)**	**Std. Error**	**Significant**	**95% Confidence interval**	
						**Lower bound**	**Upper bound**
BMI	Rarely	Never use	0.156	0.093	0.233	−0.06	0.38
	Weekly	Never use	−0.017	0.099	0.997	−0.25	0.22
	Daily	Never use	0.122	0.105	0.525	−0.13	0.37
Depression level	Rarely	Never use	0.035	0.096	0.970	−0.19	0.26
	Weekly	Never use	−0.186	0.102	0.174	−0.43	0.06
	Daily	Never use	−0.141	0.108	0.431	−0.40	0.12

* The mean difference is significant at the 0.05 level.
